# Poly[[μ_2_-acetato-aquadi-μ_3_-isonicotinato-dysprosium(III)silver(I)] perchlorate]

**DOI:** 10.1107/S1600536809046637

**Published:** 2009-11-18

**Authors:** Li-Cai Zhu

**Affiliations:** a School of Chemistry and Environment, South China Normal University, Guangzhou 510631, People’s Republic of China

## Abstract

In the title three-dimensional heterometallic complex, {[AgDy(C_6_H_4_NO_2_)_2_(C_2_H_3_O_2_)(H_2_O)]ClO_4_}_*n*_, the Dy(III) ion is eight-coordinated by four O atoms from four different isonicotinate ligands, three O atoms from two different acetate ligands and one O atom of water mol­ecule. The two-coordinate Ag^I^ ion is bonded to two N atoms from two different isonicotinate anions. These metal coordination units are connected by bridging isonicotinate and acetate ligands, generating a three-dimensional network. The coordinated water mol­ecules link the carboxyl­ate group and the acetate ligand by O—H⋯O hydrogen bonding. The perchlorate anion is disordered over two sites with site occupancy factors 0.508 (12) and 0.492 (12) and the methyl group of the acetate ligand is disordered over two positions of equal occupancy.

## Related literature

For the applications of lanthanide–transition metal heterometallic complexes with bridging multifunctional organic ligands in ion exchange, magnetism, bimetallic catalysis and as luminescent probes, see: Cheng *et al.* (2006 ▶); Kuang *et al.* (2007 ▶); Peng *et al.* (2008 ▶); Zhu *et al.* (2009 ▶).
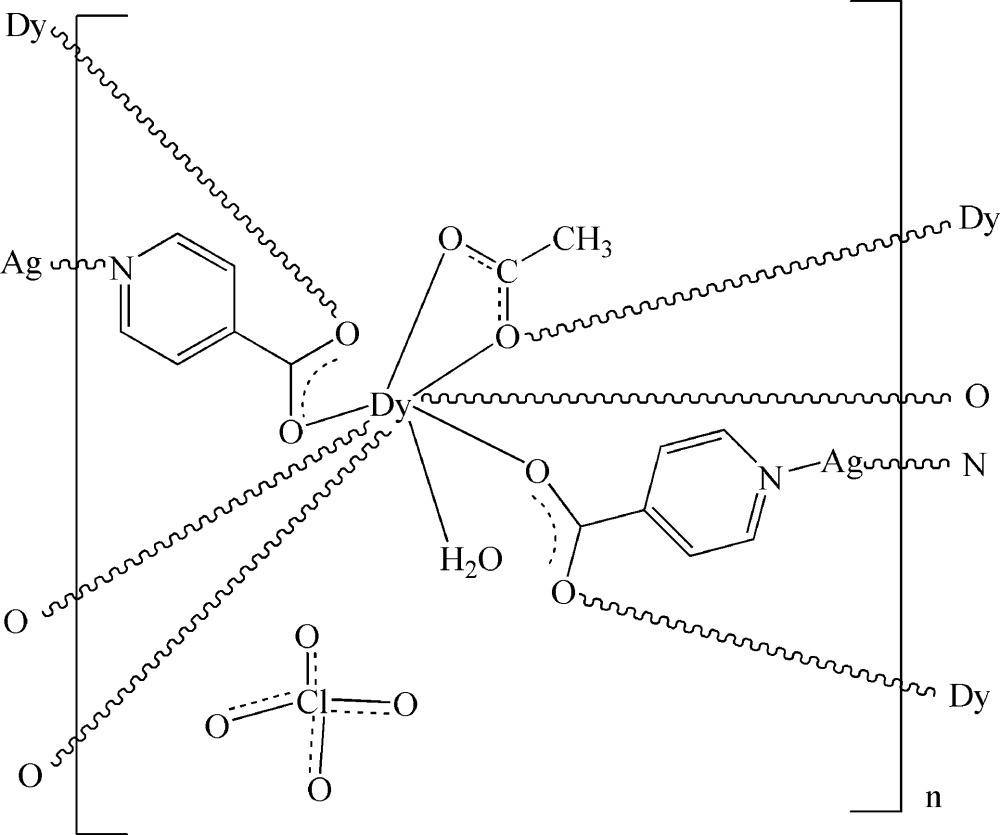


## Experimental

### 

#### Crystal data

[AgDy(C_6_H_4_NO_2_)_2_(C_2_H_3_O_2_)(H_2_O)]ClO_4_
*M* *_r_* = 691.08
Monoclinic, 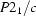

*a* = 16.1682 (15) Å
*b* = 15.1020 (14) Å
*c* = 7.9846 (7) Å
β = 92.845 (1)°
*V* = 1947.2 (3) Å^3^
*Z* = 4
Mo *K*α radiation
μ = 5.01 mm^−1^
*T* = 296 K
0.23 × 0.20 × 0.19 mm

#### Data collection

Bruker APEXII area-detector diffractometer
Absorption correction: multi-scan (*SADABS*; Sheldrick, 1996 ▶) *T*_min_ = 0.328, *T*_max_ = 0.386
9904 measured reflections
3486 independent reflections
3112 reflections with *I* > 2σ(*I*)
*R*_int_ = 0.025

#### Refinement

*R*[*F* ^2^ > 2σ(*F* ^2^)] = 0.023
*wR*(*F* ^2^) = 0.056
*S* = 1.04
3486 reflections
322 parameters
158 restraints
H atoms treated by a mixture of independent and constrained refinement
Δρ_max_ = 0.66 e Å^−3^
Δρ_min_ = −0.76 e Å^−3^

### 

Data collection: *APEX2* (Bruker, 2004 ▶); cell refinement: *SAINT* (Bruker, 2004 ▶); data reduction: *SAINT*; program(s) used to solve structure: *SHELXS97* (Sheldrick, 2008 ▶); program(s) used to refine structure: *SHELXL97* (Sheldrick, 2008 ▶); molecular graphics: *XP* in *SHELXTL* (Sheldrick, 2008 ▶); software used to prepare material for publication: *SHELXL97*.

## Supplementary Material

Crystal structure: contains datablocks I, global. DOI: 10.1107/S1600536809046637/zq2015sup1.cif

Structure factors: contains datablocks I. DOI: 10.1107/S1600536809046637/zq2015Isup2.hkl

Additional supplementary materials:  crystallographic information; 3D view; checkCIF report

## Figures and Tables

**Table 1:** Hydrogen-bond geometry (Å, °)

*D*—H⋯*A*	*D*—H	H⋯*A*	*D*⋯*A*	*D*—H⋯*A*
O1*W*—H2*W*⋯O2^i^	0.79 (3)	2.21 (4)	2.925 (4)	150 (5)
O1*W*—H1*W*⋯O5^ii^	0.79 (3)	2.05 (4)	2.813 (4)	161 (5)

Symmetry codes: (i) 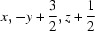; (ii) 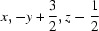.

**Fig. 1.**
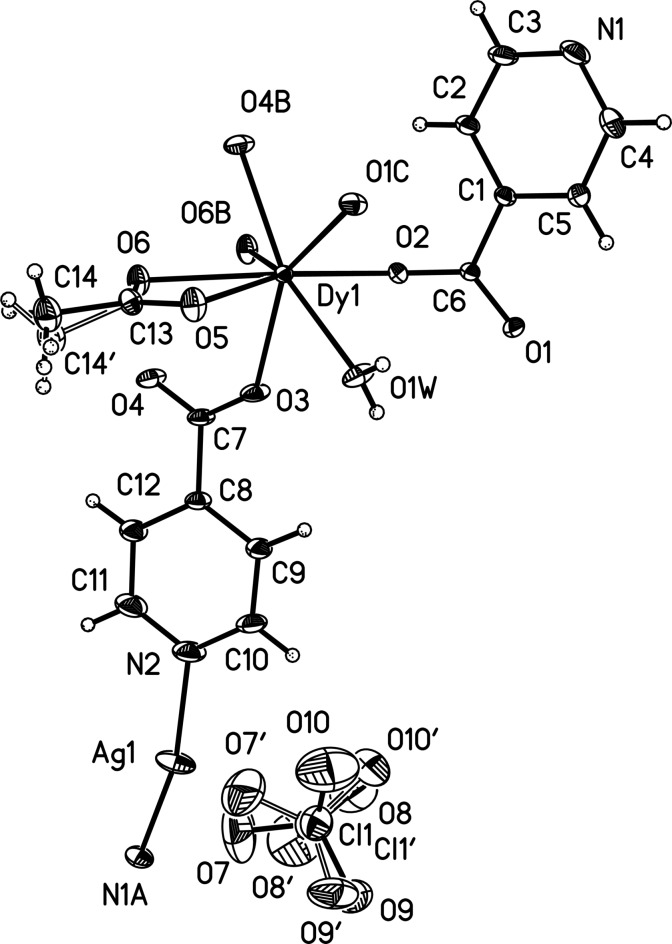
The molecular structure showing the atomic-numbering scheme and displacement ellipsoids drawn at the 30% probability level. Symmetry codes: (A) 1 + x, 1.5 - y, -1/2 + z; (B) 1 - x, 1 - y, 2 - z; (C) x, 1.5 - y, 1/2 + z.

**Fig. 2.**
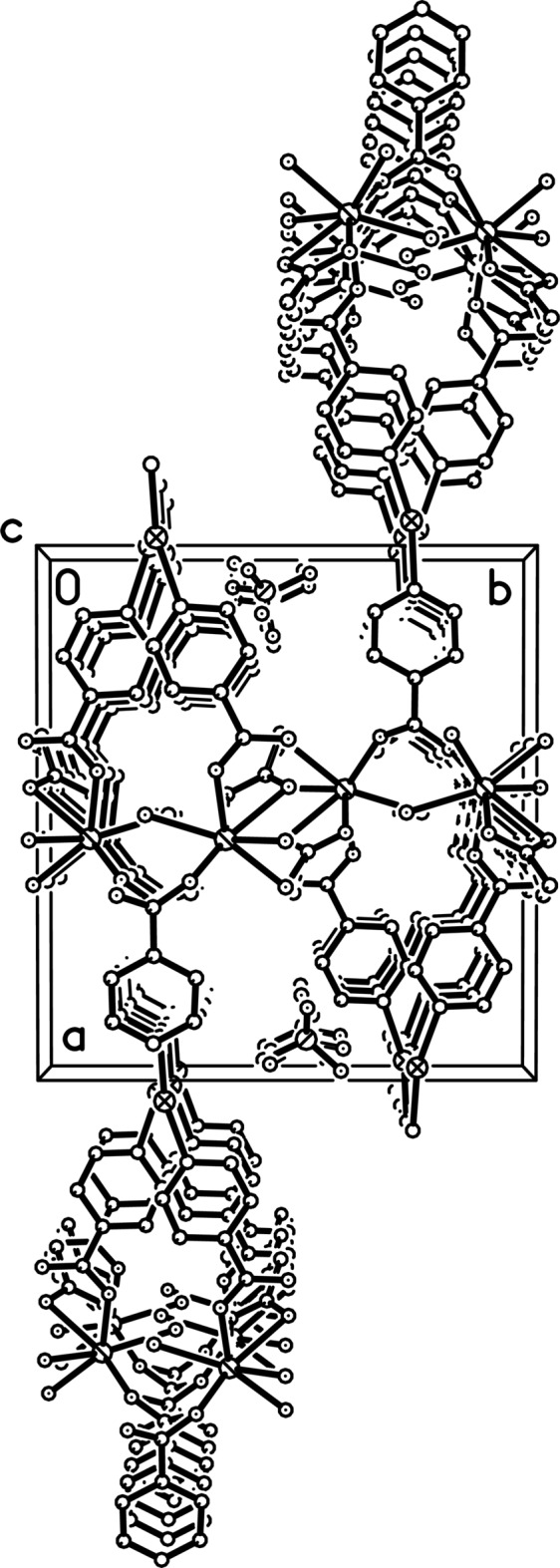
A view of the three-dimensional structure of the title compound. Hydrogen atoms are omitted for clarity.

## supplementary crystallographic information

### Comment

In the past few years, lanthanide-transition metal heterometallic complexs with bridging multifunctionnal organic ligands are of increasing interest, not only because of their impressive topological structures, but also due to their versatile applications in ion exchange, magnetism, bimetallic catalysis and luminescent probe(Cheng *et al.*, 2006; Kuang *et al.*, 2007; Peng *et al.*, 2008; Zhu *et al.*, 2009). As an extension of this research, the structure of the title compound, a new heterometallic coordination polymer, (I), has been determined which is presented in this article.

In the title compound (Fig. 1), there are one Dy(III) ion, one Ag(I) ion, two halves of acetate ligand, two isonicotinate ligands, one coordinated water molecule, and one perchlorate anion in the asymmetric unit. Each Dy(III) ion is eight-coordinated by four O atoms from four different isonicotinate ligands [Dy—O distances ranging from 2.297 (3) to 2.348 (3) Å], and three O atoms from two different acetate ligands [Dy—O distances ranging from 2.394 (3) to 2.484 (3) Å], and one O atom of water molecule [Dy—O distances 2.414 (3) Å]. The O—Dy—O bond angles are in the range from 52.70 (10) to 155.05 (10) °. The Dy center can be described as having a bicapped trigonal prism coordination geometry. The two-coordinate Ag(I) ion is bonded to two N atoms from two different isonicotinate anions [Ag—N distances 2.163 (4) Å]. Thus the Ag(I) ion is in a somewhat linear configuration with N1—Ag1—N2 angle 165.40 (17) °. These metal coordination units are connected by bridging isonicotinate and acetate ligands, generating a three-dimensional network (Fig. 2).The coordinated water molecules link the carboxylate group and acetate ligand by O—H···O hydrogen bonding (Table 1). The perchlorate anion is disordered over two sites with site occupancy factors 0.508 (12) and 0.492 (12). The methyl group of the acetate ligand is disordered over two positions of equal occupancy (0.5:0.5).

### Experimental

A mixture of AgNO_3_(0.057 g, 0.33 mmol), Dy_2_O_3_(0.116 g, 0.33 mmol), isonicotinic acid (0.164 g, 1.33 mmol), CH_3_COONa(0.057 g, 0.7 mmol), H_2_O(7 ml), and HClO_4_(0.257 mmol)(pH 2) was sealed in a 20 ml Teflon-lined reaction vessel at 443 K for 6 days then slowly cooled to room temperature. The product was collected by filtration, washed with water and air-dried. Colorless block crystals suitable for X-ray analysis were obtained.

### Refinement

H atoms bonded to C atoms were positioned geometrically and refined as riding, with C—H = 0.93 or 0.96 Å and *U*_iso_(H) = 1.2 or 1.5 *U*_eq_(C). H atoms of water molecules were found from difference Fourier maps and refined isotropically with a restraint of O—H = 0.82 Å. The perchlorate anion is disordered over two sites with site occupancy factors 0.508 (12) and 0.492 (12). The methyl group of the acetate ligand is disordered over two positions of equal occupancy (0.5:0.5).

### Crystal data

**Table:**

[AgDy(C_6_H_4_NO_2_)_2_(C_2_H_3_O_2_)(H_2_O)]ClO_4_	*F*(000) = 1316
*M**_r_* = 691.08	*D*_x_ = 2.357 Mg m^−^^3^
Monoclinic, *P*2_1_/*c*	Mo *K*α radiation, λ = 0.71073 Å
Hall symbol: -P 2ybc	Cell parameters from 5589 reflections
*a* = 16.1682 (15) Å	θ = 2.7–27.8°
*b* = 15.1020 (14) Å	µ = 5.01 mm^−^^1^
*c* = 7.9846 (7) Å	*T* = 296 K
β = 92.845 (1)°	Block, colorless
*V* = 1947.2 (3) Å^3^	0.23 × 0.20 × 0.19 mm
*Z* = 4	

### Data collection

**Table:**

Bruker APEXII area-detector diffractometer	3486 independent reflections
Radiation source: fine-focus sealed tube	3112 reflections with *I* > 2σ(*I*)
graphite	*R*_int_ = 0.025
φ and ω scan	θ_max_ = 25.2°, θ_min_ = 1.9°
Absorption correction: multi-scan (*SADABS*; Sheldrick, 1996)	*h* = −15→19
*T*_min_ = 0.328, *T*_max_ = 0.386	*k* = −18→17
9904 measured reflections	*l* = −8→9

### Refinement

**Table:**

Refinement on *F*^2^	Primary atom site location: structure-invariant direct methods
Least-squares matrix: full	Secondary atom site location: difference Fourier map
*R*[*F*^2^ > 2σ(*F*^2^)] = 0.023	Hydrogen site location: inferred from neighbouring sites
*wR*(*F*^2^) = 0.056	H atoms treated by a mixture of independent and constrained refinement
*S* = 1.03	*w* = 1/[σ^2^(*F*_o_^2^) + (0.0236*P*)^2^ + 3.5237*P*] where *P* = (*F*_o_^2^ + 2*F*_c_^2^)/3
3486 reflections	(Δ/σ)_max_ = 0.002
322 parameters	Δρ_max_ = 0.66 e Å^−^^3^
158 restraints	Δρ_min_ = −0.76 e Å^−^^3^

### Special details

**Table:**

Geometry. All e.s.d.'s (except the e.s.d. in the dihedral angle between two l.s. planes) are estimated using the full covariance matrix. The cell e.s.d.'s are taken into account individually in the estimation of e.s.d.'s in distances, angles and torsion angles; correlations between e.s.d.'s in cell parameters are only used when they are defined by crystal symmetry. An approximate (isotropic) treatment of cell e.s.d.'s is used for estimating e.s.d.'s involving l.s. planes.
Refinement. Refinement of *F*^2^ against ALL reflections. The weighted *R*-factor *wR* and goodness of fit *S* are based on *F*^2^, conventional *R*-factors *R* are based on *F*, with *F* set to zero for negative *F*^2^. The threshold expression of *F*^2^ > σ(*F*^2^) is used only for calculating *R*-factors(gt) *etc*. and is not relevant to the choice of reflections for refinement. *R*-factors based on *F*^2^ are statistically about twice as large as those based on *F*, and *R*- factors based on ALL data will be even larger.

### Fractional atomic coordinates and isotropic or equivalent isotropic displacement parameters (Å^2^)

**Table:**

	*x*	*y*	*z*	*U*_iso_*/*U*_eq_	Occ. (<1)
Dy1	0.454540 (11)	0.616178 (12)	1.04948 (2)	0.01852 (7)	
Ag1	0.97316 (3)	0.73989 (4)	0.60071 (7)	0.06665 (17)	
O1	0.35829 (18)	0.81592 (19)	0.7190 (4)	0.0300 (7)	
O2	0.38143 (17)	0.68117 (19)	0.8281 (4)	0.0263 (7)	
O3	0.5762 (2)	0.6330 (2)	0.9080 (5)	0.0401 (9)	
O4	0.63060 (19)	0.49745 (19)	0.8632 (5)	0.0403 (9)	
O5	0.5347 (2)	0.6149 (2)	1.3147 (4)	0.0360 (8)	
O6	0.54781 (19)	0.4970 (2)	1.1607 (4)	0.0320 (7)	
O1W	0.4979 (2)	0.7687 (2)	1.0758 (4)	0.0358 (8)	
H1W	0.518 (3)	0.795 (3)	1.002 (6)	0.054*	
H2W	0.475 (3)	0.800 (3)	1.139 (6)	0.054*	
N1	0.0943 (2)	0.7527 (3)	0.9981 (6)	0.0404 (10)	
N2	0.8576 (3)	0.6967 (3)	0.6991 (6)	0.0500 (12)	
C1	0.2516 (3)	0.7494 (3)	0.8695 (5)	0.0231 (9)	
C2	0.2116 (3)	0.6710 (3)	0.9112 (6)	0.0312 (11)	
H1	0.2372	0.6165	0.8967	0.037*	
C3	0.1336 (3)	0.6753 (3)	0.9741 (7)	0.0388 (12)	
H2	0.1072	0.6229	1.0008	0.047*	
C4	0.1333 (3)	0.8284 (4)	0.9589 (7)	0.0446 (13)	
H3	0.1068	0.8821	0.9760	0.054*	
C5	0.2109 (3)	0.8294 (3)	0.8945 (6)	0.0335 (11)	
H4	0.2358	0.8828	0.8680	0.040*	
C6	0.3367 (2)	0.7495 (3)	0.8008 (5)	0.0202 (9)	
C7	0.6332 (3)	0.5809 (3)	0.8637 (6)	0.0303 (10)	
C8	0.7113 (3)	0.6231 (3)	0.8058 (6)	0.0293 (10)	
C9	0.7156 (3)	0.7123 (3)	0.7613 (7)	0.0410 (13)	
H7	0.6693	0.7486	0.7668	0.049*	
C10	0.7894 (3)	0.7458 (4)	0.7088 (8)	0.0489 (15)	
H8	0.7917	0.8053	0.6791	0.059*	
C11	0.8533 (3)	0.6108 (4)	0.7457 (9)	0.0568 (17)	
H5	0.9009	0.5763	0.7427	0.068*	
C12	0.7818 (3)	0.5719 (3)	0.7975 (7)	0.0428 (13)	
H6	0.7810	0.5123	0.8264	0.051*	
C13	0.5697 (3)	0.5411 (3)	1.2898 (5)	0.0276 (10)	
C14	0.6271 (11)	0.5015 (17)	1.421 (3)	0.045 (4)	0.50
H14A	0.6719	0.5418	1.4467	0.068*	0.50
H14B	0.6487	0.4468	1.3804	0.068*	0.50
H14C	0.5976	0.4905	1.5203	0.068*	0.50
C14'	0.6444 (10)	0.5127 (17)	1.393 (3)	0.045 (4)	0.50
H14D	0.6503	0.5488	1.4915	0.068*	0.50
H14E	0.6926	0.5191	1.3282	0.068*	0.50
H14F	0.6385	0.4518	1.4246	0.068*	0.50
Cl1	0.9165 (8)	0.9620 (9)	0.7618 (15)	0.0633 (8)	0.492 (12)
O7	0.9742 (9)	0.8934 (9)	0.785 (2)	0.138 (7)	0.492 (12)
O8	0.8752 (9)	0.9496 (8)	0.6037 (14)	0.105 (5)	0.492 (12)
O9	0.9544 (10)	1.0465 (9)	0.761 (2)	0.074 (5)	0.492 (12)
O10	0.8556 (9)	0.9600 (10)	0.8849 (19)	0.139 (7)	0.492 (12)
Cl1'	0.9216 (7)	0.9553 (8)	0.7502 (15)	0.0633 (8)	0.508 (12)
O7'	0.9307 (9)	0.8895 (9)	0.8748 (17)	0.125 (6)	0.508 (12)
O8'	0.9565 (10)	0.9226 (10)	0.6054 (17)	0.139 (6)	0.508 (12)
O9'	0.9612 (10)	1.0334 (9)	0.809 (2)	0.090 (6)	0.508 (12)
O10'	0.8355 (6)	0.9715 (7)	0.720 (2)	0.101 (5)	0.508 (12)

### Atomic displacement parameters (Å^2^)

**Table:**

	*U*^11^	*U*^22^	*U*^33^	*U*^12^	*U*^13^	*U*^23^
Dy1	0.01576 (11)	0.01481 (11)	0.02554 (12)	0.00081 (7)	0.00650 (8)	0.00006 (8)
Ag1	0.0261 (2)	0.0917 (4)	0.0846 (4)	−0.0148 (2)	0.0267 (2)	0.0028 (3)
O1	0.0256 (16)	0.0230 (16)	0.0424 (19)	0.0016 (13)	0.0128 (14)	0.0096 (14)
O2	0.0190 (15)	0.0247 (15)	0.0357 (18)	0.0039 (12)	0.0055 (13)	0.0049 (13)
O3	0.0302 (19)	0.0227 (17)	0.071 (3)	−0.0018 (13)	0.0332 (18)	−0.0015 (16)
O4	0.0337 (18)	0.0193 (16)	0.070 (2)	−0.0035 (14)	0.0288 (17)	−0.0028 (16)
O5	0.044 (2)	0.0315 (18)	0.0319 (18)	0.0101 (15)	−0.0040 (15)	−0.0092 (14)
O6	0.0376 (18)	0.0270 (16)	0.0304 (17)	0.0082 (14)	−0.0078 (14)	−0.0062 (14)
O1W	0.049 (2)	0.0228 (17)	0.038 (2)	−0.0094 (15)	0.0213 (17)	−0.0029 (14)
N1	0.023 (2)	0.049 (3)	0.051 (3)	0.0021 (18)	0.0138 (19)	−0.003 (2)
N2	0.028 (2)	0.050 (3)	0.074 (3)	−0.009 (2)	0.022 (2)	−0.002 (2)
C1	0.017 (2)	0.028 (2)	0.024 (2)	0.0005 (17)	0.0038 (17)	0.0033 (17)
C2	0.020 (2)	0.026 (2)	0.049 (3)	−0.0004 (18)	0.011 (2)	0.000 (2)
C3	0.025 (2)	0.037 (3)	0.056 (3)	−0.006 (2)	0.017 (2)	−0.001 (2)
C4	0.033 (3)	0.040 (3)	0.062 (4)	0.013 (2)	0.014 (3)	−0.002 (3)
C5	0.027 (2)	0.025 (2)	0.050 (3)	0.0046 (19)	0.012 (2)	0.004 (2)
C6	0.016 (2)	0.020 (2)	0.024 (2)	−0.0002 (16)	0.0040 (17)	0.0002 (17)
C7	0.023 (2)	0.028 (2)	0.041 (3)	−0.0069 (19)	0.016 (2)	−0.001 (2)
C8	0.023 (2)	0.026 (2)	0.041 (3)	−0.0048 (18)	0.014 (2)	−0.003 (2)
C9	0.025 (3)	0.029 (3)	0.070 (4)	0.000 (2)	0.017 (2)	0.004 (2)
C10	0.032 (3)	0.036 (3)	0.081 (4)	−0.010 (2)	0.021 (3)	0.007 (3)
C11	0.026 (3)	0.047 (3)	0.100 (5)	0.000 (2)	0.019 (3)	−0.004 (3)
C12	0.027 (3)	0.031 (3)	0.073 (4)	0.001 (2)	0.017 (3)	−0.001 (3)
C13	0.026 (2)	0.032 (2)	0.025 (2)	0.0036 (19)	−0.0008 (19)	−0.0022 (19)
C14	0.046 (6)	0.045 (5)	0.044 (6)	0.006 (5)	−0.012 (5)	−0.003 (4)
C14'	0.046 (6)	0.045 (5)	0.044 (6)	0.006 (5)	−0.012 (5)	−0.003 (4)
Cl1	0.0616 (15)	0.0502 (16)	0.0776 (16)	0.0036 (11)	−0.0019 (11)	−0.0024 (12)
O7	0.145 (10)	0.105 (8)	0.162 (11)	0.076 (7)	−0.030 (8)	−0.017 (7)
O8	0.132 (10)	0.108 (8)	0.072 (7)	−0.039 (7)	−0.042 (7)	0.005 (6)
O9	0.068 (8)	0.060 (7)	0.094 (8)	−0.011 (6)	0.012 (6)	−0.003 (6)
O10	0.135 (10)	0.151 (10)	0.136 (10)	−0.038 (8)	0.054 (8)	−0.009 (8)
Cl1'	0.0616 (15)	0.0502 (16)	0.0776 (16)	0.0036 (11)	−0.0019 (11)	−0.0024 (12)
O7'	0.140 (10)	0.120 (9)	0.112 (8)	−0.019 (7)	−0.017 (7)	0.066 (7)
O8'	0.163 (10)	0.142 (9)	0.119 (9)	0.012 (8)	0.063 (8)	−0.035 (7)
O9'	0.074 (8)	0.082 (9)	0.115 (10)	−0.034 (7)	0.006 (7)	−0.038 (7)
O10'	0.055 (6)	0.088 (7)	0.160 (10)	0.004 (5)	−0.008 (6)	0.008 (7)

### Geometric parameters (Å, °)

**Table:**

Dy1—O2	2.297 (3)	C2—C3	1.382 (6)
Dy1—O4^i^	2.328 (3)	C2—H1	0.9300
Dy1—O3	2.330 (3)	C3—H2	0.9300
Dy1—O1^ii^	2.348 (3)	C4—C5	1.380 (6)
Dy1—O6^i^	2.394 (3)	C4—H3	0.9300
Dy1—O1W	2.414 (3)	C5—H4	0.9300
Dy1—O5	2.428 (3)	C7—C8	1.509 (6)
Dy1—O6	2.484 (3)	C8—C12	1.381 (6)
Dy1—C13	2.842 (4)	C8—C9	1.395 (6)
Dy1—Dy1^i^	3.9005 (5)	C9—C10	1.381 (6)
Ag1—N2	2.163 (4)	C9—H7	0.9300
Ag1—N1^iii^	2.163 (4)	C10—H8	0.9300
O1—C6	1.256 (5)	C11—C12	1.379 (7)
O1—Dy1^iv^	2.348 (3)	C11—H5	0.9300
O2—C6	1.272 (5)	C12—H6	0.9300
O3—C7	1.275 (5)	C13—C14	1.490 (9)
O4—C7	1.261 (5)	C13—C14'	1.490 (9)
O4—Dy1^i^	2.328 (3)	C14—H14A	0.9600
O5—C13	1.269 (5)	C14—H14B	0.9600
O6—C13	1.263 (5)	C14—H14C	0.9600
O6—Dy1^i^	2.394 (3)	C14'—H14D	0.9600
O1W—H1W	0.79 (3)	C14'—H14E	0.9600
O1W—H2W	0.79 (3)	C14'—H14F	0.9600
N1—C3	1.348 (6)	Cl1—O7	1.399 (12)
N1—C4	1.350 (7)	Cl1—O8	1.411 (12)
N1—Ag1^v^	2.163 (4)	Cl1—O9	1.416 (11)
N2—C10	1.335 (7)	Cl1—O10	1.426 (12)
N2—C11	1.351 (7)	Cl1'—O8'	1.401 (12)
C1—C5	1.394 (6)	Cl1'—O7'	1.408 (12)
C1—C2	1.398 (6)	Cl1'—O9'	1.411 (11)
C1—C6	1.506 (5)	Cl1'—O10'	1.420 (11)
			
O2—Dy1—O4^i^	104.83 (12)	C2—C1—C6	121.9 (4)
O2—Dy1—O3	89.71 (12)	C3—C2—C1	119.1 (4)
O4^i^—Dy1—O3	138.72 (10)	C3—C2—H1	120.4
O2—Dy1—O1^ii^	85.79 (11)	C1—C2—H1	120.4
O4^i^—Dy1—O1^ii^	74.38 (10)	N1—C3—C2	122.6 (4)
O3—Dy1—O1^ii^	146.22 (10)	N1—C3—H2	118.7
O2—Dy1—O6^i^	77.05 (10)	C2—C3—H2	118.7
O4^i^—Dy1—O6^i^	72.23 (11)	N1—C4—C5	122.6 (4)
O3—Dy1—O6^i^	73.89 (11)	N1—C4—H3	118.7
O1^ii^—Dy1—O6^i^	136.70 (11)	C5—C4—H3	118.7
O2—Dy1—O1W	78.20 (12)	C4—C5—C1	119.2 (4)
O4^i^—Dy1—O1W	148.29 (11)	C4—C5—H4	120.4
O3—Dy1—O1W	71.91 (11)	C1—C5—H4	120.4
O1^ii^—Dy1—O1W	74.40 (11)	O1—C6—O2	124.5 (4)
O6^i^—Dy1—O1W	137.44 (11)	O1—C6—C1	118.2 (3)
O2—Dy1—O5	155.05 (10)	O2—C6—C1	117.3 (3)
O4^i^—Dy1—O5	91.77 (13)	O4—C7—O3	126.4 (4)
O3—Dy1—O5	89.81 (13)	O4—C7—C8	116.7 (4)
O1^ii^—Dy1—O5	80.85 (11)	O3—C7—C8	116.9 (4)
O6^i^—Dy1—O5	126.52 (10)	C12—C8—C9	118.5 (4)
O1W—Dy1—O5	77.96 (12)	C12—C8—C7	119.0 (4)
O2—Dy1—O6	149.85 (10)	C9—C8—C7	122.5 (4)
O4^i^—Dy1—O6	73.52 (11)	C10—C9—C8	119.0 (5)
O3—Dy1—O6	74.95 (11)	C10—C9—H7	120.5
O1^ii^—Dy1—O6	121.22 (11)	C8—C9—H7	120.5
O6^i^—Dy1—O6	73.84 (12)	N2—C10—C9	122.8 (5)
O1W—Dy1—O6	119.46 (12)	N2—C10—H8	118.6
O5—Dy1—O6	52.70 (10)	C9—C10—H8	118.6
O2—Dy1—C13	169.94 (11)	N2—C11—C12	123.1 (5)
O4^i^—Dy1—C13	83.17 (13)	N2—C11—H5	118.4
O3—Dy1—C13	80.23 (13)	C12—C11—H5	118.4
O1^ii^—Dy1—C13	102.45 (12)	C11—C12—C8	118.8 (5)
O6^i^—Dy1—C13	100.12 (12)	C11—C12—H6	120.6
O1W—Dy1—C13	98.28 (13)	C8—C12—H6	120.6
O5—Dy1—C13	26.41 (11)	O6—C13—O5	118.8 (4)
O6—Dy1—C13	26.36 (11)	O6—C13—C14	120.1 (11)
O2—Dy1—Dy1^i^	114.40 (7)	O5—C13—C14	120.6 (11)
O4^i^—Dy1—Dy1^i^	68.41 (7)	O6—C13—C14'	119.0 (11)
O3—Dy1—Dy1^i^	70.39 (7)	O5—C13—C14'	121.4 (12)
O1^ii^—Dy1—Dy1^i^	140.97 (7)	C14—C13—C14'	15.6 (17)
O6^i^—Dy1—Dy1^i^	37.71 (7)	O6—C13—Dy1	60.8 (2)
O1W—Dy1—Dy1^i^	139.94 (8)	O5—C13—Dy1	58.3 (2)
O5—Dy1—Dy1^i^	88.82 (7)	C14—C13—Dy1	177.5 (10)
O6—Dy1—Dy1^i^	36.13 (7)	C14'—C13—Dy1	166.7 (9)
C13—Dy1—Dy1^i^	62.43 (9)	C13—C14—H14A	109.5
N2—Ag1—N1^iii^	165.40 (17)	C13—C14—H14B	109.5
C6—O1—Dy1^iv^	149.1 (3)	C13—C14—H14C	109.5
C6—O2—Dy1	138.1 (3)	C13—C14'—H14D	109.5
C7—O3—Dy1	134.9 (3)	C13—C14'—H14E	109.5
C7—O4—Dy1^i^	139.1 (3)	H14D—C14'—H14E	109.5
C13—O5—Dy1	95.3 (3)	C13—C14'—H14F	109.5
C13—O6—Dy1^i^	160.5 (3)	H14D—C14'—H14F	109.5
C13—O6—Dy1	92.8 (3)	H14E—C14'—H14F	109.5
Dy1^i^—O6—Dy1	106.16 (12)	O7—Cl1—O8	107.5 (10)
Dy1—O1W—H1W	123 (4)	O7—Cl1—O9	112.5 (10)
Dy1—O1W—H2W	119 (4)	O8—Cl1—O9	107.4 (10)
H1W—O1W—H2W	113 (5)	O7—Cl1—O10	111.9 (11)
C3—N1—C4	118.2 (4)	O8—Cl1—O10	107.5 (10)
C3—N1—Ag1^v^	122.7 (3)	O9—Cl1—O10	109.7 (10)
C4—N1—Ag1^v^	119.1 (3)	O8'—Cl1'—O7'	107.6 (10)
C10—N2—C11	117.6 (4)	O8'—Cl1'—O9'	111.8 (10)
C10—N2—Ag1	125.7 (4)	O7'—Cl1'—O9'	109.0 (10)
C11—N2—Ag1	116.5 (3)	O8'—Cl1'—O10'	110.8 (10)
C5—C1—C2	118.3 (4)	O7'—Cl1'—O10'	107.9 (10)
C5—C1—C6	119.8 (4)	O9'—Cl1'—O10'	109.6 (10)

Symmetry codes: (i) −*x*+1, −*y*+1, −*z*+2; (ii) *x*, −*y*+3/2, *z*+1/2; (iii) *x*+1, −*y*+3/2, *z*−1/2; (iv) *x*, −*y*+3/2, *z*−1/2; (v) *x*−1, −*y*+3/2, *z*+1/2.

### Hydrogen-bond geometry (Å, °)

**Table:**

*D*—H···*A*	*D*—H	H···*A*	*D*···*A*	*D*—H···*A*
O1W—H2W···O2^ii^	0.79 (3)	2.21 (4)	2.925 (4)	150 (5)
O1W—H1W···O5^iv^	0.79 (3)	2.05 (4)	2.813 (4)	161 (5)

Symmetry codes: (ii) *x*, −*y*+3/2, *z*+1/2; (iv) *x*, −*y*+3/2, *z*−1/2.
